# Investigation of pesticides on honey bee carbonic anhydrase inhibition

**DOI:** 10.1080/14756366.2020.1835885

**Published:** 2020-10-20

**Authors:** Ercan Soydan, Ahmet Can Olcay, Gürkan Bilir, Ömer Taş, Murat Şentürk, Deniz Ekinci, Claudiu T. Supuran

**Affiliations:** a Faculty of Agriculture, Department of Agricultural Biotechnology, Ondokuz Mayıs University, Samsun, Turkey; b Pharmacy Faculty, Department of Biochemistry, Agri Ibrahim Cecen University, Agri, Turkey; c Neurofarba Department, University of Florence, Firenze, Italy

**Keywords:** Carbonic anhydrase, bee, pesticides, inhibition, pyrethroids

## Abstract

Carbonic anhydrase (CA, EC 4.2.1.1) plays crucial physiological roles in many different organisms, such as in pH regulation, ion transport, and metabolic processes. CA was isolated from the European bee *Apis mellifera* (AmCA) spermatheca and inhibitory effects of pesticides belonging to various classes, such as carbamates, thiophosphates, and pyrethroids, were investigated herein. The inhibitory effects of methomyl, oxamyl, deltamethrin, cypermethrin, dichlorodiphenyltrichloroethane (DDT) and diazinon on AmCA were analysed. These pesticides showed effective *in vitro* inhibition of the enzyme, at sub-micromolar levels. The IC_50_ values for these pesticides ranged between of 0.0023 and 0.0385 μM. The CA inhibition mechanism with these compounds is unknown at the moment, but most of them contain ester functionalities which may be hydrolysed by the enzyme with the formation of intermediates that can either react with amino acid residues or bid to the zinc ion from the active site.

## 1. Introduction

Pesticides are chemical compounds that are used against various pests as a biological control agent. Some pesticides are persistent organic contaminants in the soil and environment. They also constitute one of the most significant causes of pollution worldwide^1^. The rapid growth of agriculture and animal processing has caused bees to be exposed to such contaminants with which they had never previously come into contact. Growing food demand has forced farmers to use more mineral fertilisers and pesticides for producing higher yields^2^. In recent decades the increasing concern about the effect of pesticides on pollinators has been expressed in the scientific literature^3^. Fort his reason, some new data were collected from laboratory and semi-field studies on the toxic effects of pesticides on bees, especially bumble bees^3^^,^^4^. A variety of articles have highlighted the significance of bees as natural pollinators not only for our crops but also for wildflowers and woodland plants, in temperate and tropical habitats^5–7^. For example, it has been reported that about 60 crop plant species may not bear growing fruit without bees as impollinators^8^; with devastating economic implications.

Carbonic anhydrases (Cas, EC 4.2.1.1) are found in almost all living organisms and control pH and CO_2_/bicarbonate levels^9^. Many different CA isoenzymes have been identified in higher vertebrates, although these enzymes are less investigated in other species, such as the arthropods, including insects^10–12^. The physiological role of CA isozymes is to promote CO_2_ to HCO_3_^−^ interconversion, thus, playing vital functions in various biochemical/physiological processes, including physiological pH regulation, gas balancing, calcification, photosynthesis, metabolism, etc^9–12^. Additionally, in vertebrates, CAs play a significant role in the eye, kidneys, central nervous system (CNS), inner ear, and many other organs, in terms of ion transfer, pH regulation, and metabolism^13–15^.

Pesticides and fungicides interfering with rainwater, irrigation water, or groundwater, plants, can inhibit particular enzymes^13–15^. In our group’s previous research, we investigated the effects of several widely used pesticides, such as tebuconazole, propoxur, carbaryl, carbofuran, simazine, and atrazine on the recently discovered bee CA, termed AmCA^15^. However, little is known about the effects of other chemical agents on this enzyme. Therefore, in this study, we purified honey bee spermatheca AmCA enzyme and analysed its interactions with pesticides and fungicides belonging to other classes.

## 2. Materials and methods

### 2.1. Chemicals

All chemicals for the affinity system were provided by Sigma-Aldrich (St. Louis, MO). Other reagents were obtained from Merck (Darmstadt, Germany).

### 2.2. Homogenate

Bee spermatheca samples were washed three times with 50 mM Tris/Sulphate (pH 7.8). Spermatecha samples taken from 200 bees were combined and homogenised with liquid nitrogen, then placed in the same buffer and centrifuged at 4 °C, 15,000 *g* for 30 min. The precipitate portion was discarded and the supernatant portion was separated and used in subsequent studies.

### 2.3. Purification of the enzyme

The enzyme was purified using Sepharose-4B-sulfanilamide affinity gel prepared by our group^15^. Aniline was used as a spacer arm in the chromatography column. The column was then equilibrated with 25 mM Tris-HCl/0.1M Na_2_SO_4_ (pH 8.7) and the gel was washed with 25 mM Tris-HCl/22 mM Na_2_SO_4_ (pH 8.7). Finally, elution was performed with 1 M NaCl/25 mM Na_2_HPO_4_ (pH 6.3). The temperature was kept at 4 °C during all experiments.

### 2.4. *In vitro* inhibition experiments for AmCA enzyme

The effects of methomyl, oxamyl, deltamethrin, dichlorodiphenyltrichloroethane (DTT), cypermethrin and diazinon on AmCA activities were assayed colorimetrically using the CO_2_ hydrase assay^16^, in triplicate at each concentration of inhibitor (ranging from 1 nM to 10 mM). Control enzyme activity was taken as 100%. An activity % versus inhibitor graph was drawn for all pesticides (Microsoft Office 2000, Excel). Pesticide concentrations that caused 50% inhibition (IC_50_) of the enzyme activity were thus obtained graphically using a regression software. The enzyme concentration in the assay system was 8.9 nM.

## 3. Results and discussion

The collapse of honeybee (*Apis mellifera*) colonies poses an important problem in many developed countries^17–20^. In fact, bee colonies are economically important for honey and wax products. The collapse of hive clones is generally related to two phenomena: (i) parasites, such as virus^21^, nosema infections^22^, mites^23^, and hive insects^24^, which attack the colony; and (ii) pesticides use, which negatively impacts the insects due to their toxicity^25^. Furthermore, such low pesticide levels can also make bees an easier target to biological infections^20–28^.

The majority of the problems in beehives faced by beekeepers are due to biological factors^27^. However, these factors are improbable to be the most important reason for the recent decrease of bumblebees in North America and Europe or the disappearing of several wild beespecies^22^^,^^28^. Agrochemicals, including pesticides, are probably the most significant factors that provoke honeybee and wild bee colonies extinction. Much research has been conducted ultimately in order to understand this problem in North America^29^, France^30^, Spain^31^ and India^32^. Such research focussed on determining the amount and prevalence of pesticide residues in honey, pollen, wax, and other different beehive matrices (e.g. combs)^29–31^. A dataset was thus established for explaining the effects of pesticide residue both on honeybees and, potentially wild bees^33^^,^^34^.

Recently, we isolated and characterised the α-CA enzyme from *Apis mellifera* spermatheca, AmCA^15^. In that study, we were able to purify the enzyme in a single step using Sepharose 4B tyrosine-sulfanilamide affinity chromatography^15^. Furthermore, pesticides such as tebuconazole, carbaryl, carbonfuran, atrazine, simazin, and propoxur were tested on the amCA enzyme and IC_50_ values were determined as 0.0030, 0.0031, 0.0087, 0.0165, 0.0273, and 0.0321 μM, respectively (Table 1)^15^. The efficiency order of pesticides AmCA inhibition was: tebuconazole > carbaryl > carbofuran > atrazine > simazine > propoxur.

**Table 1.** AmCA inhibition data from our previous study^15^.

Inhibitor	IC_50_ (μM)
Tebuconazole	0.0030
Carbaryl	0.0031
Carbonfuran	0.0087
Atrazine	0.0165
Simazin	0.0273
Propoxur	0.0321

**Figure 1.**
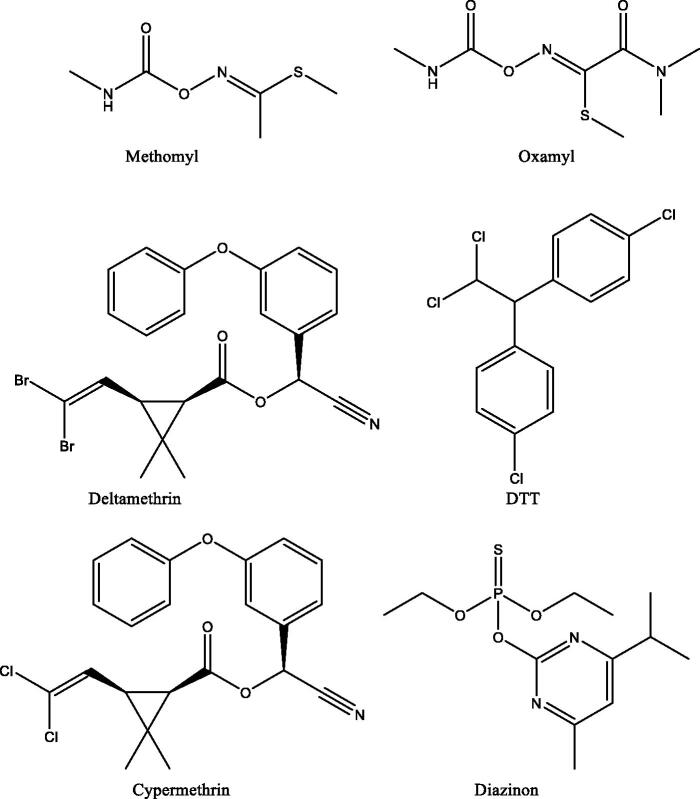
Chemical structures of the pesticides tested as inhibitors of AmCA in this study.

Here we report the inhibitory effects of 6 pesticides belonging to various classes (Fıgure 1) on AmCA. Indeed, carbamates, thiophosphates, and pyrethroids were considered as potential CA inhibitors in the present study (Figure 1).

Besides the enzyme purification already reported in the previous work^15^, the inhibitory effects of six different pesticides on honeybee spermatheca CA and their IC_50_ parameters were investigated here. The activity of AmCA was inhibited by pesticides shown in Figure 1 at low micromolar concentrations. Indeed, the IC_50_ values were determined to be: 0.0023 ± 0.0001, 0.0025 ± 0.0001, 0.0028 ± 0.0001, 0.0034 ± 0.0001, 0.0078 ± 0.0003, and 0.0385 ± 0.0012 μM for methomyl, oxamyl, deltamethrin, dichlorodiphenyltrichloroethane (DTT), cypermethrin, and diazinon, respectively (Table 2).

**Table 2.** AmCA inhibition data for new pesticides shown in Figure 1.

Inhibitor	IC_50_ (μM)
Methomyl	0.0023 ± 0.0001
Oxamyl	0.0025 ± 0.0001
Deltamethrin	0.0028 ± 0.0001
DDT	0.0034 ± 0.0001
Cypermethrin	0.0078 ± 0.0003
Diazinon	0.0385 ± 0.0012

It should be mentioned that a detailed study on pesticide residues in bees was conducted by Sanchez-Bayo and Goka^20^. In this study, topical LD_50_ and oral LD_50_ values of pesticides against honey bees and bumblebees were reported^20^. The topical lethal dose (LD_50_) rates found in this study^20^ were as follows: deltamethrin 0.02 μg/bee, cypermethrin 0.03 μg/bee, methomyl 0.49 μg/bee, and diazinon 0.38 μg/bee. In the same study, the oral dose rates were as follows: cypermethrin 0.06 μg/bee, methomyl 3.38 μg/bee, diazinon 0.21 μg/bee, and DDT 5.08 μg/bee^20^. Compared to this literature results^20^, the results obtained in the present study show a low level of IC_50_ for the inhibition of AmCA with pesticides presented in Figure 1 and widely used in agriculture (Table 2). Thus, our results may prove that pesticides as those presented in Figure 1 may induce a strong inhibition on honey bee spermatheca CA enzyme. It is not known at the moment whether this inhibition may lead to physiological consequences but studies are ongoing in the field in our laboratories.

There has been a great interest in recent studies focussed on the effects of several classes of chemicals on CA enzymes^35–37^. CA is a classical metalloenzyme, whose isozymes have significant roles in many tissues, that has been characterized and purified from several organisms, including animals^35–37^. A huge number of pollutants, such as metals, acids, bases, and other toxic compounds^38^ are being mixed in water sources and also in the atmosphere, which increasingly damages our environment. The potent AmCA inhibitory effects of compounds shown in Figure 1 may represent a potential explanation of why bees (domestic and wild ones) are under pressure worldwide with an increasing level of extinction of many diverse such insect species.

This also brings us to the possible mechanism of action of these pesticides investigated here. The carbamates, thiophosphates, and pyrethroids investigated here (Figure 1) possess ester bonds which can be hydrolysed by the esterase activity of the α-CAs. In fact, it has been thoroughly documented that these enzymes are esterases/thioesterases/selenoestearses with carboxylic, phosphoric, thiocarboxylic, and even selenol esters^39–42^. Only DDT does not have this functionality, but this compound was reported by Bitman et al. to act as a CAI in the 70s^43^.

The two carbamates from Figure 1, methomyl, and oxamyl can be substrates of CAs which may hydrolyse their ester/thioester bonds with the formation of small molecules which can bind to the metal centre (acetate, methyl-thiol, or Me_2_N–COCOOH in the case of the second carbamate). The pyrethroids may also be hydrolysed at their ester functionality, with the generation of carboxylic acids and alcohols which were shown to act as CAIs^12^^,^^13^^,^^36^. Diazinon has thiophosphate functionalities which were shown to be hydrolysed by the esterase activity of CAs in previous work from this laboratory, leading to suicide inhibitors of the enzyme^39^. However, these hypotheses should be verified by X-ray crystallography, one of the most powerful techniques useful to assess CA inhibition mechanisms, especially the innovative ones. This technique also has its weak points, especially when used in an inattentive manner. The best example is the report by Liljas’ group that cyanide and cyanate do not coordinate the metal ion from CA active site^44^. Subsequent work from other laboratories showed those data to be false, as both cyanate and cyanide were observed coordinated to the metal ion, as most other anion inhibitors investigated to date^45^^,^^46^. Furthermore, cyanate was also shown to be a suicide substrate that can be hydrolysed by the CA activity with the formation of carbamate^45^. However, bitter and dubious comments from the above-mentioned crystallography group continued even 30 years later^47^.

## 4. Conclusions

AmCA was purified from *Apis mellifera* spermatheca by affinity chromatography. Pesticides such as methomyl, oxamyl, deltamethrin, and DDT showed inhibition effects, comparable to those of cypermethrin and diazinon. The IC_50_ values were determined as 0.0023 ± 0.0001, 0.0025 ± 0.0001, 0.0028 ± 0.0001, 0.0034 ± 0.0001, 0.0078 ± 0.0003 and 0.0385 ± 0.0012 μM, respectively (Table 2). Our results showed that pesticides inhibit AmCA activity with the following order: methomyl > oxamyl > deltamethrin > DDT > cypermethrin > diazinon. Our findings indicate these pesticides to act as potent inhibitors of AmCA, which might cause undesirable biological effects in bees, by disrupting their acid–base regulation as well as salt transport. Further studies are warranted in order to understand whether these inhibition data are relevant for the significant diminution of domestic and wild bee species worldwide. Several studies on bees as well as other organisms showed that sperm life depends on different parameters, among which the pH. It was observed that sperm loses its vitality if the pH level increases. This supports the relevance of the present findings. However, the data presented here need a careful *in vivo* (or in the hive) validation.

## References

[1] Ekinci D, Senturk M, Interactions of fungicides and pesticides with specific enzymes. In: Carisse O. ed. Fungicides. London, UK: In-Tech Open Publisher, 2010: 38–404.

[2] Sattarı SZ, Bouwman AF, Martınez Rodríguez R, et al. Negative global phosphorus budgets challenge sustainable intensification of grasslands. Nat Commun 2016;7:10696. 26882144 10.1038/ncomms10696 PMC4757762

[3] Osborne JL. Ecology: bumblebees and pesticides. Nature 2012;491:43–5. 23086148 10.1038/nature11637

[4] Marletto F, Patetta A, Manino A. Laboratory assessment of pesticide toxicity to bumble bees. Bull Insectology 2003;56:155–8.

[5] Klein A-M, Vaissière BE, Cane JH, et al. Importance of pollinators in changing landscapes for world crops. P Roy Soc B-Biol Sci 2007;274:303–13. 10.1098/rspb.2006.3721 PMC1702377 17164193

[6] Allen-Wardell G, Bernhardt P, Bitner R, et al. The potential consequences of pollinator declines on the conservation of biodiversity and stability of food crop yields. Conserv Biol 1998;12:8–17.

[7] Potts SG, Biesmeijer JC, Kremen C, et al. Global pollinator declines: trends, impacts and drivers. Trends Ecol Evol 2010;25:345–53. 20188434 10.1016/j.tree.2010.01.007

[8] Heard TA. The role of stingless bees in crop pollination. Annu Rev Entomol 1999;44:183–206. 15012371 10.1146/annurev.ento.44.1.183

[9] (a) Supuran CT. Carbonic anhydrases: novel therapeutic applications for inhibitors and activators. Nature Rev Drug Discov 2008;7:168. 18167490 10.1038/nrd2467

[10] (a) Demirdag R, Comakli V, Senturk M, et al. Characterization of carbonic anhydrase from sheep kidney and effects of sulfonamides on enzyme activity. Bioorg Med Chem 2013;21:1522–5. 22974493 10.1016/j.bmc.2012.08.018

[11] (a) Ekinci D, Kurbanoglu NI, Salamci E, et al. Carbonic anhydrase inhibitors: inhibition of human and bovine isoenzymes by benzenesulphonamides, cyclitols and phenolic compounds. J Enzyme Inhib Med Chem 2012;27:845–8. 21999604 10.3109/14756366.2011.621122

[12] (a) Özdemir ZÖ, Şentürk M, Ekinci D. Inhibition of mammalian carbonic anhydrase isoforms I, II and VI with thiamine and thiamine-like molecules. J Enzyme Inhib Med Chem 2013;28:316–9. 22145674 10.3109/14756366.2011.637200

[13] a)Ceyhun SB, Sentürk M, Yerlikaya E, et al. Purification and characterization of carbonic anhydrase from the teleost fish *Dicentrarchus labrax* (European seabass) liver and toxicological effects of metals on enzyme activity. Environ Toxicol Pharmacol 2011;32:69–74. 21787732 10.1016/j.etap.2011.03.013

[14] Ceyhun SB, Şentürk M, Ekinci D, et al. Deltamethrin attenuates antioxidant defense system and induces the expression of heat shock protein 70 in rainbow trout. Comp Biochem Phys C 2010;152:215–23. 10.1016/j.cbpc.2010.04.008 20417719

[15] Soydan E, Guler A, Biyik S, et al. Carbonic anhydrase from *Apis mellifera*: purification and inhibition by pesticides. J Enzyme Inhib Med Chem 2017;32:47–50. 28090787 10.1080/14756366.2016.1232255 PMC6009862

[16] Wilbur KM, Anderson NG. Electrometric and colorimetric determination of carbonic anhydrase. J Biol Chem 1948;176:147–54. 18886152

[17] Wang D, Hu J, Bobulescu IA, et al. A sperm-specific Na+/H + exchanger (sNHE) is critical for expression and in vivo bicarbonate regulation of the soluble adenylyl cyclase (sAC). Proc Natl Acad Sci USA 2007;104:9325–30. 17517652 10.1073/pnas.0611296104 PMC1890493

[18] Pettis JS, Rice N, Joselow K, et al. Colony failure linked to low sperm viability in honey bee (*Apis mellifera*) queens and an exploration of potential causative factors. PLoS One 2016;11:e0155833. 26863438 10.1371/journal.pone.0147220 PMC4749221

[19] Paynter E, Harvey M, Welch M, et al. Insights into the molecular basis of long-term storage and survival of sperm in the honeybee (*Apis mellifera*). Sci Rep 2017;7:40236. 28091518 10.1038/srep40236 PMC5238380

[20] Sanchez-Bayo F, Goka K. Pesticide residues and bees – a risk assessment. PLoS One 2014;9:e94482. 24718419 10.1371/journal.pone.0094482 PMC3981812

[21] Cox-Foster DL, Conlan S, Holmes EC, et al. A metagenomic survey of microbes in honey bee colony collapse disorder. Science 2007;318:283–7. 17823314 10.1126/science.1146498

[22] Cameron SA, Lozier JD, Strange JP, et al. Patterns of widespread decline in North American bumble bees. Proc Natl Acad Sci USA 2011;108:662–7. 21199943 10.1073/pnas.1014743108 PMC3021065

[23] Thompson H, Ball R, Brown M, et al. Varroa destructor resistance to pyrethroid treatments in the United Kingdom. Bull Insect 2003;56:175–81.

[24] Underwood RM, vanEngelsdorp D. Colony Collapse Disorder: have we seen this before? Bee Culture 2007;135:13–15.

[25] Maini S, Medrzycki P, Porrini C. The puzzle of honey bee losses: a brief review. Bull Insect 2010;63:153–60.

[26] Vidau C, Diogon M, Aufauvre J, et al. Exposure to sublethal doses of fipronil and thiacloprid highly increases mortality of honeybees previously infected by *Nosema ceranae*. PLoS One 2011;6:e21550. 21738706 10.1371/journal.pone.0021550 PMC3125288

[27] Williams GR, Tarpy DR, vanEngelsdorp D, et al. Colony collapse disorder in context. BioEssays 2010;32:845–6. 20730842 10.1002/bies.201000075 PMC3034041

[28] Goulson D, Lye GC, Darvill B. Decline and conservation of bumble bees. Annual Rev Entomol 2008;53:191–208. 17803456 10.1146/annurev.ento.53.103106.093454

[29] Stoner KA, Eitzer BD. Using a hazard quotient to evaluate pesticide residues detected in pollen trapped from honey bees (*Apis mellifera*) in Connecticut. PLoS One 2013;8:e77550. 24143241 10.1371/journal.pone.0077550 PMC3797043

[30] Chauzat MP, Carpentier P, Martel AC, et al. Influence of pesticide residues on honey bee (Hymenoptera: Apidae) colony health in France. Environ Entomol 2009;38:514–23. 19508759 10.1603/022.038.0302

[31] Bernal J, Garrido-Bailon E, Nozal MJd, et al. Overview of pesticide residues in stored pollen and their potential effect on bee colony (*Apis mellifera*) losses in Spain. J Econ Entomol 2010;103:1964–71. 21309214 10.1603/ec10235

[32] Choudhary A, Sharma DC. Dynamics of pesticide residues in nectar and pollen of mustard (*Brassica juncea* (L.) Czern.) grown in Himachal Pradesh (India). Environ Monit Assess 2008;144:143–50. 17952621 10.1007/s10661-007-9952-3

[33] Thompson HM. Assessing the exposure and toxicity of pesticides to bumblebees (*Bombus* sp.). Apidologie 2001;32:305–21.

[34] Greig-Smith PW, Thompson HM, Hardy AR, et al. Incidents of poisoning of honeybees (*Apis mellifera*) by agricultural pesticides in Great Britain 1981–1991. Crop Prot 1994;13:567–81.

[35] (a) Ekinci D, Sentürk M, Küfrevioğlu Öİ. Salicylic acid derivatives: synthesis, features and usage as therapeutic tools. Expert Opin Ther Pat 2011;21:1831–41. 22098318 10.1517/13543776.2011.636354

[36] (a) Ekinci D, Ceyhun SB, Şentürk M, et al. Characterization and anions inhibition studies of an α-carbonic anhydrase from the teleost fish *Dicentrarchus labrax*. Bioorg Med Chem 2011;19:744–8. 21211980 10.1016/j.bmc.2010.12.033

[37] (a) Dizdaroglu Y, Albay C, Arslan T, et al. Design, synthesis and molecular modelling studies of some pyrazole derivatives as carbonic anhydrase inhibitors. J Enzyme Inhib Med Chem 2020;35:289–97. 31797703 10.1080/14756366.2019.1695791 PMC6896446

[38] Bielmyer GK, Brix KV, Grosell A. Is Cl- protection against silver toxicity due to chemical speciation? Aquat Toxicol 2008;87:81–7. 18304659 10.1016/j.aquatox.2008.01.004

[39] (a) Innocenti A, Scozzafava A, Parkkila S, et al. Investigations of the esterase, phosphatase, and sulfatase activities of the cytosolic mammalian carbonic anhydrase isoforms I, II, and XIII with 4-nitrophenyl esters as substrates. Bioorg Med Chem Lett 2008;18:2267–71. 18353640 10.1016/j.bmcl.2008.03.012

[40] (a) Lopez M, Vu H, Wang CK, et al. Promiscuity of carbonic anhydrase II. Unexpected ester hydrolysis of carbohydrate-based sulfamate inhibitors. J Am Chem Soc 2011;133:18452–62. 21958118 10.1021/ja207855c

[41] Tanc M, Carta F, Scozzafava A, Supuran CT. α-Carbonic anhydrases possess thioesterase activity. ACS Med Chem Lett 2015;6:292–5. 25815148 10.1021/ml500470b PMC4360143

[42] Angeli A, Carta F, Donnini S, et al. Selenolesterase enzyme activity of carbonic anhydrases. Chem Commun 2020;56:4444–7. 10.1039/d0cc00995d 32195510

[43] Bitman J, Cecil HC, Fries GF. DDT-induced inhibition of avian shell gland carbonic anhydrase: a mechanism for thin eggshells. Science 1970;168:594–6. 4985323 10.1126/science.168.3931.594

[44] Lindahl M, Svensson LA, Liljas A. Metal poison inhibition of carbonic anhydrase. Proteins 1993;15:177–82. 8441752 10.1002/prot.340150207

[45] Supuran CT, Conroy CW, Maren TH. Is cyanate a carbonic anhydrase substrate? Proteins 1997;27:272–8. 9061790 10.1002/(sici)1097-0134(199702)27:2<272::aid-prot12>3.0.co;2-j

[46] West D, Pinard MA, Tu C, et al. Human carbonic anhydrase II-cyanate inhibitor complex: putting the debate to rest. Acta Crystallogr F Struct Biol Commun 2014;70:1324–7. 25286933 10.1107/S2053230X14018135 PMC4188073

[47] Jonsson BH, Liljas A. Perspectives on the classical enzyme carbonic anhydrase and the search for inhibitors. Biophys J 2020;119:1275–80. 32910900 10.1016/j.bpj.2020.08.020 PMC7567974

